# Supplementary material for: Enhancing blood availability in Latin America: A study on public perceptions and barriers to blood donation in Guatemala during the COVID-19 pandemic
Source: PLOS Glob Public Health. 2025 Feb 11;5(2):e0003437. doi: 10.1371/journal.pgph.0003437 (PMC11813101; doi:10.1371/journal.pgph.0003437)
Supplement: S1 Appendix — (DOCX) [file pgph.0003437.s003.docx]

**S1 Appendix.** Translated survey instrument in English

| 1. Gender | (   ) Male  (  ) Female | |
| --- | --- | --- |
| 1. How old are you? | (   ) <18  (   ) 19-29  (   ) 30-39 | (   ) 40-49  (   ) 50-59  (   ) 60+ |
| 1. Where do you live? | (   ) Guatemala City  (   ) Sacatepéquez  (   ) Huehuetenango  (   ) San Marcos  (   ) Quetzaltenango  (   ) Chimaltenango  (   ) Santa Rosa  (   ) Alta Verapaz  (   ) Escuintla | (   ) Quiché  (   ) Sololá  (   ) Izabal  (   ) Chiquimula  (   ) Baja Verapaz  (   ) Jutiapa  (   ) Petén  (   ) I do not live in Guatemala |
| 1. What is the highest level of education that you have completed? | (   ) Elementary School  (   ) High School  (   ) Bachelors | (   ) Master  (   ) Doctorate |
| 1. Have you donated blood in the past? | (   ) Yes    (   ) No | |
| 1. If you have donated blood in the past, where did you donate? *(Select all that apply)* | (   ) I donated to a family member/friend/referred donation  (   ) I donated at a donation campaign from the Red Cross  (   ) I donated at a DonaGuate campaign  (   ) I have never donated before  (   ) Others *(please specify in the comments)* | |
| 1. If you have never donated blood, is there a specific reason? *(Select all that apply)* | (   ) I have donated before  (   ) I am scared of needles  (   ) I do not know where to donate  (   ) I cannot donate for medical reasons  (   ) I do not trust in the hygiene of the donation facilities  (   ) I do not trust how donated blood is used  (   ) I am afraid of the secondary effects of donating blood  (   ) I would only donate if it was for someone I know  (   ) I am afraid to become infected with diseases through needles  (   ) I cannot donate due to religious beliefs  (   ) My work schedule complicates going to donate blood  (   ) Donation centers are not close to me  (   ) I never thought of it before  (   ) I have a rare type of blood and prefer to donate for emergencies only  (   ) Other *(please specify in the comments)* | |
| 1. Do you know where you can donate blood in Guatemala? | (   ) Yes    (   ) No | |
| 1. Are you willing to donate blood? | (   ) Yes, but only for emergencies of family, friends, and people I know  (   ) Yes, but only for emergencies – it does not matter who receives the blood  (   ) Yes, I would like to be a frequent donor (1 time per year)  (   ) I am not interested in donating blood | |
| 1. If you were to donate blood, do you care who receives your blood? | (   ) Yes    (   ) No | |
| 1. What would facilitate or motivate you to donate blood? | [Open text] | |
