# Supplementary material for: Enhancing blood availability in Latin America: A study on public perceptions and barriers to blood donation in Guatemala during the COVID-19 pandemic
Source: PLOS Glob Public Health. 2025 Feb 11;5(2):e0003437. doi: 10.1371/journal.pgph.0003437 (PMC11813101; doi:10.1371/journal.pgph.0003437)
Supplement: Attachment — Submitted filename: Response to Reviewer_PLOS GB.docx [file pgph.0003437.s004.docx]

**PGPH-D-24-01168
Enhancing Blood Availability in Latin America: A Study on Public Perceptions and Barriers to Blood Donation in Guatemala during the COVID-19 Pandemic
PLOS Global Public Health**

Esteemed editorial team,

We greatly appreciate your insightful comments and suggestions regarding our manuscript “Enhancing Blood Availability in Latin America: A Study on Public Perceptions and Barriers to Blood Donation in Guatemala during the COVID-10 Pandemic”. Below, we have provided responses to each of your comments and detailed the revisions made to address them.

| **Reviewer Comment** | **Author Comments** | **Manuscript Revisions** |
| --- | --- | --- |
| ***Reviewer 1:*** | | |
| 1) “Introduction: The title of the essay, the abstract, and the introduction do not seem to be well-coordinated. What exactly is the goal of the analysis? Please clarify this point further in the abstract and introduction.” | We thank the reviewer for pointing this out. We have revised the abstract and the introduction to better align with the title and the study’s goals. | The abstract and the introduction now better describe the focus and aim of the study, as reflected in the study’s title, which is primarily identifying the population factors influencing blood donation behavior during the first year of the COVID-19 pandemic in Guatemala. (see p. 3-4) |
| 2) “Introduction: I miss seeing more literature on the motivations and barriers of donors from other Latin American countries in the introduction. Isn't there a larger body of research on this topic?” | We agree with this comment. Our team conducted multiple literature reviews on this topic, both in English and Spanish, finding a concerning small number of scientific manuscripts. Most of the information on this topic, comes from larger international or regional organizations, such as the WHO or the Red Cross. | The introduction has been revised to reflect the limited available scientific information on the motivators and barriers to blood donation in the Latin American context. |
| 3) “Introduction: "Moreover, these nations commonly grapple with a range of challenges that contribute to the limited availability of blood products…” The list in this sentence could be clearer with semicolons or separate points.” | We thank the reviewer for the grammatical observation. | The sentence in the introduction has been corrected. (see p.2 for the revised version of the introduction) |
| 4) “Introduction: “urgent urgency” may be simplified to “urgent need”” | We thank the reviewer for the grammatical observation. | This sentence was also reformatted as a result of the revisions made in the previous comments. (see p 2-3 for the revised version) |
| 5) “Methods: It would greatly help the readers to know more about the survey. How many people were contacted? What were the age limits for participation? Did all population groups have access to the survey? Were there any population groups that could not participate due to a language barrier? What was the response rate of the survey? To what extent does the sample accurately reflect the population? If no information is available on this, it would still be helpful to know how the participants were selected.” | We appreciate the reviewer’s comments on the study methodology. The survey was designed in Spanish, the official language of Guatemala. Although it is the official language, there are 24 distinct indigenous languages spoken in Guatemala. The survey unfortunately did not reach this significant portion of the population. In addition, given the snowball methodology, it was impossible to estimate how many people were reached by the survey and as a result, no response rate could be estimated. Only the complete responses from those living in Guatemala at the time of the survey and those older than 18 years of age were included int he analysis. | The methods section “Survey distribution” has been revised to reflect the comments made by the editor, primarily how participants were identified, how the survey was distributed among the population, who had access to it and what limitations it had. (see p.3-4) |
| 6) “Methods: What does it mean if someone has already donated? When was this donation? Were these whole blood donations? How exactly was the question phrased?” | We thank the reviewer for asking this important information. As presented in the translated survey instrument (Appendix 1), the question in the survey asked participants “Have you donated blood in the past?” with the option to answer only Yes or No to this question. The question did not address what type of blood donation or when this occurred. (see p. 17) | We have clarified that previous blood donors refer to participants who have donated at least once prior to the survey. (see p. 4) |
| 7) “Methods: I am a bit confused. Under 'statistical analysis,' it states that a review board was involved. Under 'ethics statements,' it says that no board was necessary. Please clarify this discrepancy.” | Thank you for noticing this error in the manuscript. This discrepancy has been corrected. The study did not require approval from an ethics review board as per institutional guidelines given that the research information security level was determined as non-sensitive. This is now clearly explained in both the Ethics Statement” section. | The Methods section “Ethics statement” has been revised to include the determination of the Longwood IRB committee and the lack of need for additional review or approval. see p.6) |
| 8) “Methods: Could you please give more information on Dona Guate?” | We thank the reviewer for this important comment. More information about DonaGuate has been added to the Methods section | This information has been added to the “Study design” section of the Methods. (see p. 4) |
| 9) “Results: Could you explain in more detail what you mean by ‘preference regarding the recipient of their donated blood’” | This referred to whether participants expressed a preference for their blood to go to a specific individual (such as family or friends) or had no preference. | This explanation has been added to the results section (see p. 7) |
| 10) “Results: “Comparing donor status (previous donors vs. never donors), male respondents exhibited a higher rate of blood donation compared to females (p < 0.001). Respondents under 29 years of age were less commonly previous donors compared to other age groups (p = 0.001), whereas those over 60 were more likely to be previous donors (p < 0.001)“ Could you please explain on which table you refer here?” | This information is reflected in Table 1. | The results has been revised to specific that the information can be found in Table 1. |
| 11) “Results: “Among previous donors (n = 610), the most common avenues for donation included referrals to family or friends (77.8%, n = 475), donations via Red Cross campaigns (10%, n = 61),…” Could you please explain on which table you refer here?” | We thank the reviewer for this observation. The information described in not listed in any of the supplemental tables, but will be included in the study database provided with the manusctipt revisions. | Please see supplemental database provided with manuscript revisions for the entire data described in the results. |
| 12) “Results: could you please explain how ‘improved access to donation centers’ could be a motivation to donate? How did you ask about motivation?” | The survey asked participants via open text what would facilitate or motivate them to donate blood (see appendix 1). This open-ended data was analyzed via thematic analysis and themes were identified, including the need reported by participants of improved access to donation centers. Figure 1 represents the frequencies for these themes. | The results section has been revised to clarify the qualitative methodology applied in the study (see p. 8) Please also refer to appendix 1 and Figure 1 for additional information. |
| 13) “Results: access to information about blood donation is the third and not the second most frequently mentioned facilitator.” | We agree with the reviewers’ comment. Initially, the themes had been divided among facilitators and motivators per the author’s judgement but later decided to include all themes as facilitators/motivators given the subjective definition of each. | The results section has been revised to reflect the most common facilitators/motivators described by each group (never donors and previous donors), now reflected in separate figures. (see p. 8 and 20) |
| 14) “Discussion: Please include a section at the beginning of the discussion summarizing the key findings of the analysis. What are the most important results?” | We thank the reviewer for this comment. An additional paragraph has been added in the discussion to include the requested information. | Please see the revised discussion to note the first paragraph which summarizes the study’s key findings. (see p. 9) |
| 15) “Discussion: Please give information on source 29” | We thank the reviewer for noting this error in the manuscript’s references. The numbering of our references was incorrect, including a repeated reference #9. | The numbering order of our references has been corrected in the manuscript (see p. 14-16) |
| 16) “In the discussion, there is little reference to the results of the survey. What are the conclusions drawn from the presented analysis? Why are blood donation rates particularly low in Guatemala?” | We thank the reviewer for his valuable commentary. We agree that the discussion includes limited reference to the results of the survey. | The discussion has been modified to align better with the results of the survey and the recommendations made to inform strategies aimed at enhancing blood availability in the country. (see p. 9-13) |
| 17) “Discussion: In the limitations section, it should be noted that most of the respondents were from Guatemala City.” | We thank the reviewer for this timely comment and agree with his suggestion. | The limitations section has been revised to reflect that most of the responses from the study were from participants located in Guatemala City. (see p. 13) |
| 18) “Figure 1 and 2: It would be helpful to see the values in percent.” | We thank the reviewer for this observation, however, would like to politely disagree with this suggestion. Figures 1 and 2 display the frequencies of the qualitative themes obtained from the open-ended responses by participants. Given that the data derived from thematic analysis focuses on the occurrence of certain ideas or topics rather than quantifiable, evenly distributed variables. Given the open nature of the responses, participants often provided information related to one or more themes in their free-text responses. In addition, this data involves subjective interpretation by two or more authors and presenting percentages can give a misleading sense of precision or overstate the representativeness of certain themes. Frequencies are reported to illustrate how often certain themes emerged within the subgroups (never donors and previous donors), without implying that these are statistically representative proportions of the population. This approach better aligns with the nature of qualitative research, which emphasizes understanding patterns, ideas, and experiences over numerical representation. | Figure 1 was divided into Figure 1a and Figure 1b representing the most common facilitators and motivators found among never donors and previous donors, respectively. We believe this representation aligns better with the discussion of the manuscript and the previous suggestions of the reviewer. (see p. 20) |
