# Supplementary material for: Enhancing blood availability in Latin America: A study on public perceptions and barriers to blood donation in Guatemala during the COVID-19 pandemic
Source: PLOS Glob Public Health. 2025 Feb 11;5(2):e0003437. doi: 10.1371/journal.pgph.0003437 (PMC11813101; doi:10.1371/journal.pgph.0003437)
Supplement: Attachment — Submitted filename: PGPH-D-24-01168R1.docx [file pgph.0003437.s005.docx]

PGPH-D-24-01168R1
Enhancing Blood Availability in Latin America: A Study on Public Perceptions and Barriers to Blood Donation in Guatemala during the COVID-19 Pandemic
PLOS Global Public Health

This manuscript deals with the transition of a country from a replacement-based model to voluntary (non-remunerated?) blood donation and the effects of the COVID pandemic. While reading the introduction I wondered about some background information about Latin America or Guatemala, that I later read in the discussion. For instance, at the end of the first paragraph of the introduction it is stated that ‘donation rates … experienced a concerning decline’. And in the last paragraph ‘with the second lowest voluntary blood donation rate’. These numbers are mentioned in the discussion. Maybe a reshuffle would improve the readability of the paper. But also, in the study design it is stated that it is a survey among the population of Guatemala, it would help the reader if here the total inhabitants would have been mentioned. At different places in the manuscript abbreviations are mentioned that have not been explained this would also be helpful.

At the end of the Statistical analysis part, it is stated that it was approved by the Harvard Longwood Campus Institutional Review Board, but at the end of the Ethics statement it states that the study did not meet the criteria for requiring approval. Does this mean that it was approved since it did not require an official approval, or can you explain this?

In the second paragraph of the discussion, it is stated that the existing shortage of voluntary donors is exacerbated by COVID-19. How is dealt with this shortage? Is this shortage also a result of the high discard rate of up to 30%? Is all the collection of blood still in transfusion centers of are there now also (separate) collection centers?

In the second paragraph on page 11 starting with Lastly, it is stated that the PAHO currently recommends a limit age for blood donation that varies between 65 and 81 years. This should be upper limit I suppose. And what is then the lower limit? The same goes for Guatemala. Furthermore, a reduction in interval between donations is suggested for increasing the supply. What is the interval now and what would you suggest to make it?

I have also a few questions about the survey itself.

The survey was conducted in August -September. In what phase was the COVID pandemic in Guatemala at that time? Where there any restrictions in place? In the second paragraph of the discussion the current pandemic is mentioned. Is it still ongoing and/or are there still restrictions?

The survey was aimed at the complete population, but only a distributed through a mailing list and through social media. How big was this list and was the approximate visiting number of their social media? If you expect to get more responses through a snowball sampling, should the survey then not be available for a linger time than three months?

In the result section previous donors (N=610) are compared to never donors (N=531)? So almost as much never donors as previous donors answered the survey. I find this very surprising given the snowball sampling procedure. I think this deserves to be explained in more detail. Furthermore, I wondered if ever donated, means that you do not yet have (many) repeat donors?

In the section about barriers and facilitators a total of 1120 respondents are mentioned out of the 1141 respondents. Does this mean that participants are allowed only one answer and/or the question is not mandatory? The mentioned barrier is this also in question 11? Or was this question only asked of never donors? In the last paragraph of the result section a smaller subgroup is mentioned. I wonder what that subgroup was.
