# Supplementary material for: Enhancing blood availability in Latin America: A study on public perceptions and barriers to blood donation in Guatemala during the COVID-19 pandemic
Source: PLOS Glob Public Health. 2025 Feb 11;5(2):e0003437. doi: 10.1371/journal.pgph.0003437 (PMC11813101; doi:10.1371/journal.pgph.0003437)
Supplement: Attachment — Submitted filename: Response to Reviewers_GBP_PLOS GPH 12.14.24.docx [file pgph.0003437.s006.docx]

**PGPH-D-24-01168R1
Enhancing Blood Availability in Latin America: A Study on Public Perceptions and Barriers to Blood Donation in Guatemala during the COVID-19 Pandemic
PLOS Global Public Health**

Esteemed editorial team,

We greatly appreciate your insightful comments and suggestions regarding our manuscript “Enhancing Blood Availability in Latin America: A Study on Public Perceptions and Barriers to Blood Donation in Guatemala during the COVID-10 Pandemic”. Below, we have provided responses to each of your comments and detailed the revisions made to address them.

| **Reviewer Comment** | **Author Comments** | **Manuscript Revisions** |
| --- | --- | --- |
| “...At the end of the first paragraph of the introduction it is stated that ‘donation rates … experienced a concerning decline’. And in the last paragraph ‘with the second lowest voluntary blood donation rate’. These numbers are mentioned in the discussion. Maybe a reshuffle would improve the readability of the paper.” | We thank the reviewer for this comment. In the latest version of the manuscript, the introduction and discussion have been modified to include this background information, in a manner that we believe improves the readability of the paper. | The introduction and discussion have been modified to reflect this and other commentary made by the reviewer. |
| “...In the study design it is stated that it is a survey among the population of Guatemala, it would help the reader if here the total inhabitants would have been mentioned” | We thank the reviewer for this observation. The total number of inhabitants in Guatemala for 2020 has been added to the methods of our manuscript. | We have included the population in Guatemala for 2020 to the study design section of the mansucript. |
| “At different places in the manuscript abbreviations are mentioned that have not been explained this would also be helpful.” | We thank the reviewer for this important observation that will improve the readability of our manuscript. We have reviewed the entire manuscript and included the full name of organizations or other abbreviations not previously explained in the manuscript. | The manuscript has been revised and all abbreviations have been explained. |
| “At the end of the Statistical analysis part, it is stated that it was approved by the Harvard Longwood Campus Institutional Review Board, but at the end of the Ethics statement it states that the study did not meet the criteria for requiring approval. Does this mean that it was approved since it did not require an official approval, or can you explain this?” | We thank the reviewer and would like to clarify this important comment. The latest version of the manuscript submitted to the journal, titled Manuscript_PLOS_GB 9.10.24, explains under the Ethics Statement that the Harvard Longwood Campus IRB determined that the study did not meet criteria for requiring IRB approval. | The sentence “This research was approved by the Harvard Longwood Campus Institutional Review Board under protocol IRB 23-0135.” which was at the end of the “statistical Analysis” section in the older version of the manuscript (Manuscript_GBP_PLOS v2.1 submitted in June 2024) has been removed in the newer version (Manuscript_PLOS_GB 9.10.24 submitted in September 2024) |
| “In the second paragraph of the discussion, it is stated that the existing shortage of voluntary donors is exacerbated by COVID-19. How is dealt with this shortage? Is this shortage also a result of the high discard rate of up to 30%? Is all the collection of blood still in transfusion centers of are there now also (separate) collection centers?” | We appreciate this important comment by the reviewer. The survey conducted in this study revealed safety and hygienic concerns by respondents during the voluntary blood donation process as one of the most common barriers for blood donation. This includes not only bloodborne infections but also COVID-19. Our study unfortunately was not designed to investigate the reasons behind the shortage of units or where the collected units are stored and there is very limited and scant public information available on this matter. We, however, believe that safety fears in the general population might have affected their willingness to attend donation centers voluntarily. This is an important issue that needs to be further investigated and we hope that this work can help in our efforts to guide the conversation with the local government and ministry of health. | The discussion has been modified to explain further the safety fears expressed by respondents that might have affected their willingness to attend blood donation centers in addition to recommendations on how to address this challenge. |
| “In the second paragraph on page 11 starting with Lastly, it is stated that the PAHO currently recommends a limit age for blood donation that varies between 65 and 81 years. This should be upper limit I suppose. And what is then the lower limit? The same goes for Guatemala. Furthermore, a reduction in interval between donations is suggested for increasing the supply. What is the interval now and what would you suggest to make it?” | We thank the reviewer for this important observation. The Pan American Health Organization has set a minimum blood donation age of 17 years old and a maximum age ranging from 65 to 81 years of age based on the health conditions of the local donor population. In Guatemala, only those younger than 55 years of age are allowed to donate, which offers an opportunity to expand the pool of donors.  In addition, donors are not allowed to donate more often than every 3 months (men) or 4 months (females) in Guatemala. The study included in our discussion by Di Angelantonio observed no significant differences in quality of life, physical activity, or cognitive function among more frequent donors. | The discuss has been modified to clarify that 65 to 81 is the recommended upper limit age recommended by the PAHO. The current time intervenal between donations recommended in Guatemala has also been added, as well as the lowest time interval proven to be safe based on our reference. |
| “The survey was conducted in August -September. In what phase was the COVID pandemic in Guatemala at that time? Where there any restrictions in place? In the second paragraph of the discussion the current pandemic is mentioned. Is it still ongoing and/or are there still restrictions?” | Thank you for your comment and the opportunity to provide further context. The survey was conducted during August–September 2020, a period when Guatemala was in a critical phase of the COVID-19 pandemic. At that time, the country was experiencing significant restrictions, including curfews, mandatory mask mandates, limited public transportation, and restrictions on public gatherings. Many healthcare services were also focused on pandemic-related care, which may have influenced general access to and attitudes towards blood donation.  In the second paragraph of the discussion, we mention the current pandemic to acknowledge its potential ongoing influence on donor behaviors and societal attitudes. While some restrictions have since been lifted, the long-term consequences of the pandemic, particularly in 2020, remain relevant to understanding the context in which this study was conducted. | The discussion has been modified toto clarify the timing, specific restrictions in place during the survey period, and the potential implications for our findings.  Please let us know if additional details are required. |
| “The survey was aimed at the complete population, but only a distributed through a mailing list and through social media. How big was this list and was the approximate visiting number of their social media? If you expect to get more responses through a snowball sampling, should the survey then not be available for a linger time than three months?” | Thank you for your comment and the opportunity to provide further clarification. After careful investigation with DonaGuate, we regret that we do not have precise information on the size of the mailing list or the number of social media visits during the study period. While exact size of the mailing list or visitor numbers to the social media platformns are unavailable, we ensured that the survey was shared multiple times across these platforms and asked participants to refer other individuals who they know to maximize visibility and engagement.  Regarding the duration of the survey, it was open for three months based on considerations of logistical feasibility and resource availability for analysis and follow-up. While extending the survey period could potentially have increased participation, previous research has shown that the majority of responses in online surveys tend to occur within the first few weeks of distribution. Additionally, the snowball sampling method allowed for an extended reach through participants sharing the survey within their networks, helping to diversify and expand participation during the set time frame.  We appreciate your suggestion and will consider extending the survey period in future studies to further maximize responses and reach. Please let us know if additional details would be helpful. | The rationale for the sampling method and duration of survey dissemination are explained under the “Survey distribution” section of the manuscript. |
| “In the result section previous donors (N=610) are compared to never donors (N=531)? So almost as much never donors as previous donors answered the survey. I find this very surprising given the snowball sampling procedure. I think this deserves to be explained in more detail. Furthermore, I wondered if ever donated, means that you do not yet have (many) repeat donors?” | We thank the reviewer for your thoughtful comment. We understand the surprise regarding the nearly equal distribution of previous donors (N=610) and never donors (N=531) given the snowball sampling procedure. This result likely reflects the broad reach of our recruitment strategy, which included both blood donation-related networks and general community outreach. While snowball sampling can sometimes lead to overrepresentation of specific groups, our efforts to disseminate the survey through diverse channels may have facilitated a balanced representation of previous and never donors.  Regarding the definition of "ever donated," it refers to individuals who have donated blood at least once in their lifetime. While this group may include some repeat donors, the survey did not specifically quantify the frequency or regularity of donations. We agree that distinguishing between first-time donors and repeat donors could provide valuable insights and will consider this in future research. | No modifications have been made to the manuscript.  Please let us know if additional clarifications are needed. |
| “In the section about barriers and facilitators a total of 1120 respondents are mentioned out of the 1141 respondents. Does this mean that participants are allowed only one answer and/or the question is not mandatory? The mentioned barrier is this also in question 11? Or was this question only asked of never donors? In the last paragraph of the result section a smaller subgroup is mentioned. I wonder what that subgroup was.” | We thank the reviewer for these observations and questions. The open-ended question included in the survey (#11 “What would facilitate or motivate you to donate blood?”) was not mandatory. Each respondent was only allowed one free-text answer. A total of 1120 responses were received and analyzed. All respondents (previous and never donors) were allowed to respond to this question as stated in the survey design.  Regarding the last sentence of the results section “A smaller subgroup also expressed hesitancy regarding the proper utilization of donated blood, expressing apprehension about potential illegal sales or unequal distribution among the population.” This subgroup refers to individuals who expressed hesitancy or specific concerns about the proper utilization of donated blood. This group represented a distinct segment of respondents with a unique perspective. We have revised the manuscript to clarify this and ensure better understanding of the results. | The last sentence of the results has been modified to improve the comprehension of the text. -> “A smaller sample of respondents also expressed hesitancy regarding the proper utilization of donated blood, expressing apprehension about potential illegal sales or unequal distribution among the population.” |
